# An Extraordinary Case of Difficult Parturition, Successfully Treated

**Published:** 1799-04

**Authors:** Thomas Archer

**Affiliations:** Harford-Town, Maryland


### *An extraordinary Case of difficult Parturition, successfully treated:—by Dr.* Thomas Archer, *of Harford-Town, Maryland.*

ON the morning of the 9th of May last, I was requested to visit a female servant of Mrs. E. who had been in labour four days. On examination with my hand to know the situation of the child, I was surprised to find that the os uteri was not dilated to more than the size of a halfpenny. It formed a thick, rigid, cartilaginous ring, not yielding nor becoming softened by the pain. The midwife in attendance informed me, that the patient was in her 30th year; that at her fifteenth year she had a prolapsus uteri, which was reduced, after it was washed in a strong decoction of white-oak bark, and dusted with powdered resin; that this was her first child; that her pains had been considerably forcing, but the intervals between them long; and that the waters had been gradually discharging for two days. From this statement of her situation, I did not doubt but her labour would be lingering. Her constitution was robust and strong. Her pulse was evidently marked with symptoms of tension or convulsive action. I let blood to eighteen ounces, directed gentle laxatives, with emollient glysters to be administered occasionally; oleaginous injections into the vagina, and the vapours of hot water were advised, as means for relaxing the os tincæ. She took a dose of opium and stramonium to procure rest, and remove unprofitable pains. I now left her to the care of the attending midwife, till the following day in the evening.

May 10th. At this time there was no perceptible alteration in the dilatation of the os uteri. The child's head could now with difficulty be felt through the os uteri, which was advancing with every pain, without dilating in the least. A few pains protruded the uterus, with its contents, without the os externum! The child was now evidently dead; which, from several well marked circumstances, had taken place about ten days previous to the present period. I dreaded the event of a case so new and alarming, and informed her mistress, there was scarcely a chance for her life; that it was possible to deliver her, but the consequences of the operation might be fatal to her. The distance of a physician rendered it impossible to call a consultation. Death appeared as the closing scene of every plan I proposed; in fact, her situation required such immediate assistance, that there was scarcely a moment for deliberation. To leave a mass within the uterus, which was hourly becoming more offensive, and to be an idle spectator of the fatal event that must ensue, was depriving her even of the chance which a doubtful and desperate remedy afforded.

I recollected having read feveral well attefted cafes, where the uterus was lacerated by its violent contraction round the body of the child in parturition. The child was fqueezed partly through the aperture into the abdomen; it was, however, delivered, and the lacerated uterus healed without any difficulty, or even the occurrence of an uncommon fymptom. The favourable event of thefe extraordinary cafes, determined me to hazard an artificial divifion of the neck of the uterus (which appeared to be lefs dangerous than a laceration), to make room for the delivery of the child. Unknown to her, or any of the attendants, excepting the midwife who held the candle (for it was now night), with a common fpear-pointed lancet, I made *three* incifions in the neck of the womb, which was very much diftended; each about two inches in length, viz. one from the uterus leading towards the urethra; one towards the perinæum; and the third towards the left labium. The pains, at this time, were not ftrong; yet they were fufficient to expel the child. After the incifions were made, the delivery was almoft inftantaneous. The umbilical chord was wrapped round the body, arm, and neck of the child. The incifions produced no pain, neither did there any hæmorrhage follow the lancet. The uterus, after the feparation of the fecundines, which came away without difficulty, contracted, and returned with but little affiftance to its priftine fituation.

The patient was now put to bed, and defired to be kept quiet. I directed anodynes to be given occafionally, and glyfters to be adminiftered, fo as to procure one or two dejections daily. Neither forenefs, pain, nor fever followed this practice, more than would have happened in any eafy, natural labour! The lochial difcharges were very inconfiderable, and not at all offenfive. She was up and walking about her room in three weeks after delivery, and is now in perfect health.

